# The most important problems and needs of rasopathy patients with a noonan syndrome spectrum disorder

**DOI:** 10.1186/s13023-023-02818-y

**Published:** 2023-07-21

**Authors:** Dagmar K. Tiemens, Lotte Kleimeier, Erika Leenders, Ellen Wingbermühle, Renee L. Roelofs, Barbara Sibbles, Floor S.M. Oostwegel, Eva Vroonland, Conny van Leeuwen, Hanneke Niessen, Paul Sonnega, Anniek Duursma, Michel A. A. P. Willemsen, Jos M. T. Draaisma, Carina A.C.M. Pittens

**Affiliations:** 1 Department of Pediatrics, Radboud Institute for Health Sciences, Amalia Children’s Hospital, Radboud university medical center, Nijmegen, The Netherlands; 2 Faculty of Earth and Life Science, Athena Institute for Research on Innovation and Communication in Health and Life Sciences, VU University, Amsterdam, The Netherlands; 3 Dutch Noonan Syndrome Foundation, Nijkerk, The Netherlands; 4 Department of Human Genetics, Radboud university medical center, Nijmegen, The Netherlands; 5 Department of Pediatrics, Erasmus MC-Sophia Children’s Hospital, Rotterdam, The Netherlands; 6 PGOsupport, Utrecht, The Netherlands; 7 Centre of Excellence for Neuropsychiatry, Vincent van Gogh Institute for Psychiatry, Venray, The Netherlands; 8 Department of Pediatrics, Donders Institute for Brain, Cognition and Behavior, Amalia Children’s Hospital Nijmegen, Nijmegen, The Netherlands; 9 Donders Institute for Brain, Cognition and Behavior, Amalia Children’s Hospital Nijmegen, Nijmegen, The Netherlands

**Keywords:** Noonan Syndrome Spectrum Disorders, Rasopathies, Ras/MAPK pathway, ERK1/2, Patient involvement, Clinical guidelines, Cognitive and behavioral functioning, Coagulation problems, Energy, Feeding problems, Pain, Musculoskeletal system, Education

## Abstract

**Background:**

Noonan syndrome spectrum disorders (NSSDs) constitute a group within the Rasopathies, and are one of the largest groups of syndromes with impact on multi-organ involvement known. The extreme variability of the clinical phenotype is, among others, due to the numerous different genes that are involved, and the differences in clinical presentation over the life span. We have studied the needs of patients and their relatives aiming to develop, evaluate and choose focus in research, medical care and policy to better meet their perspectives.

**Methods:**

Using the participatory and interactive Dialogue method, 80 patients and relatives mentioned 53 different problems or needs (topics) that were categorized into eight themes. These themes and the topics within each theme, were subsequently prioritized by putting them in order of importance methodologically.

**Results:**

The four highest prioritized themes were: (1) Physical problems (non-musculoskeletal related); (2) Social, emotional and behavioral problems; (3) Cognitive functioning and information processing; and (4) Problems related to the musculoskeletal system. Nineteen out of the 53 topics were physical problems. According to the total group of respondents, the top 3 prioritized topics within theme 1 were coagulation problems, heart problems, and feeding problems. Also data stratified by age groups, phenotype (NS and other NSSDs) and gender showed some remarkable results. For instance, feeding problems were prioritized as the most important topic of the highest prioritized theme, according to patients aged 0–12 years. Also feeding problems show a significant difference in its prioritization according to female patients (2) compared to male patients (7). On the other hand, heart problems were not mentioned in the top three prioritized topics in the youngest age groups, although heart problems are generally considered most important for patients with NSSD.

**Conclusions:**

With our results we underline the importance of methodologically inventorying the needs of NSSD patients, not only at the group level, but to also focus on specific needs according to e.g. age, phenotype and gender. For instance, it is remarkable that both the current Clinical Guidelines and the Noonan Syndrome diagnostic criteria give little to no attention to feeding problems, though our results indicate that, to the youngest patients, these problems have top priority. A similar situation appears to apply to the clinical management of e.g. coagulation, neuropsychological and musculoskeletal problems (like physiotherapy or occupational therapy) and to a need for (educational) tools to support patients at school or at work. Our study may help to shape targeted (clinical) management, research and policy inside and outside medical (research) institutes and shed light on the complex phenotypes of NSSDs, the families’ and patients’ perspectives on the everyday consequences of the many different problems, as well as their needs.

## Introduction

Noonan syndrome spectrum disorders (NSSDs) are a group of genetically and phenotypically related conditions, resembling Noonan syndrome, caused by heterozygous pathogenic germline variants in genes within the Ras/mitogen-activated protein kinase (Ras/MAPK) signalling pathway. The clinical presentation can be extremely variable. In this group, the syndrome with the highest prevalence is Noonan syndrome (NS; OMIM 163,950). Other NSSDs are Noonan syndrome with multiple lentigines (NSML; OMIM 151,100), Noonan syndrome with loose anagen hair (NS-LAH; OMIM 607,721), Noonan syndrome-like disorder (CBL; OMIM 613,563), cardio-facio-cutaneous syndrome (CFCS; OMIM 115,150), and Costello syndrome (CS; OMIM 218,040) [1]. Currently, at least 19 genes involved in NSSDs have been identified, of which the most common are *PTPN11, SOS1, SOS2, KRAS, HRAS, RAF1, RIT1*, and *LZTR1* [2].

As the Ras/MAPK pathway regulates the cell cycle, differentiation, growth, and cell senescence in almost every cell in the body, children and adults with one of these syndromes generally develop multi-organ symptoms. These symptoms can vary from mild to severe or even life-threatening. As a consequence, the variety of not only the physical, but also the neuropsychological and social issues that can be encountered in NSSDs is wide. Choosing focus in the large amount of potential topics on research, policy and quality of care, both in and outside health care institutions, therefor is complicated in NSSDs.

### Objective

For these reasons, we have studied the needs of patients and their relatives aiming to develop and evaluate research, medical care and policy to better meet their perspectives. Gaining insight in the needs of patients and their relatives has become increasingly important over the last decades for a wide variety of arguments [3–5]. As knowledge of patients’ experience may give a novel view on current knowledge or can be complementary to professional knowledge, patients’ perspective may add substantial information (*substantial argument)* [6]. Also, as patients are ‘end users’ of developed policy and knowledge, they are entitled to be involved in decision making that is affecting them (*normative argument*). Additionally, an active role of patients can lead to an increase in its support and implementation (*political argument*) [7, 8]. In other words, integrating patients’ experiences could provide for a wider perspective, and thereby contribute to increased quality and relevance of research and policy development [9, 10].

Currently only few research projects have been identified with a more or less active engagement in one of the phases of research development, medical care or policy development concerning patients with NSSD, their relatives, family support groups or patient representative organizations [11]. However, the needs of patients with NSSDs and their relatives has not been structurally explored. Therefore, to gain insight in the needs of patients and their relatives in a structured way, we have executed the first two phases of the Dialogue Model developed in The Netherlands [5]. The Dialogue Model is based on participatory and interactive methodologies and the Interactive learning and action approach [3, 4, 6], and has now been used in over 15 separate well studied patient agenda projects [e.g. 5, 10, 12]. The information we obtained can be used to guide decision-making in the process of research, care and policy development, based on what patients with NSSDs and their relatives in the Netherlands (and probably also in other countries) need the most.

## Materials and methods

### Project management

The research activities were carried out between September 2019 and April 2020 (partly in the period of the pandemic SARS-CoV-2 (COVID)). A project group of stakeholder representatives – consisting of patient representatives, researchers and health professionals – was formed. Patients and relatives (proxies) were approached by the Dutch Noonan Syndrome Foundation by newsletters, presentations at two annual national meetings, and informal digital canals. In Phase 2 of the project, patients and relatives known at the Radboudumc were also informed about this study by letter.

### Research design

In this study we describe the following phases of the Dialogue Model: (1) initiation end exploration resulting in a first inquiring questionnaire and (2) consultation and prioritization resulting in a second larger and targeted questionnaire. In the final questionnaire, patients and relatives were asked to make their voices heard and to indicate specifically what they thought were the most important topics to tackle, for example in the field of care, scientific research, better information or participation in society.

The final questionnaire was designed using Survey Monkey and enabled individual participants to weigh the various topics within themes by prioritizing the listed topics as most important (score 1), second most important (score 2) etcetera. This way each topic gained a score per theme. The respondents were asked to prioritize the eight themes in relation to each other. In the final part of the questionnaire respondents were asked to mention topics that they thought were missing in the questionnaire.

The numerically designed patient specific questionnaire was statistically analyzed, resulting in a general prioritization of the themes compared to each other and a list of most important topics within each theme. Additionally differences in prioritization were stratified according to age groups, clinical diagnose (phenotype) (NS versus other NSSDs) and gender.

***Phase 1*** Initiation and exploration (September – October 2019).

Several informal meetings (not qualitatively analyzed) resulted in a first short questionnaire that was designed to allow patients and their relatives to put forward the issues and topics they considered most impactful in relation to NSDDs without restrictions. The questionnaire was submitted during one of the annual patient and caregiver meetings (September 2019), organized by the Dutch NS Foundation. The number of respondents was 13 patients and 47 relatives. Then, the mentioned topics were structured and framed into a first proposal of patient specific themes.

***Phase 2***: Consultation and Prioritizing (October 2019 – April 2020).

The objective of developing the second more targeted questionnaire was three-fold: (1) to select and list the most urgent topics, (2) to select and name the themes, and (3) to cluster the topics within the most appropriate theme. Resources for finalizing the topics per theme were (1) the results of the first survey (2) document analysis of the Dutch Medical Guideline Noonan Syndromes [13] and (3) consultation by the project group. This survey was improved in four Delphi rounds with the project group members. Then, the resulted survey was tested by a caregiver and a NSSD patient and finalized with their feedback.

In January 2020, patients and their relatives were invited to participate in the questionnaire through various channels of the Dutch Noonan Syndrome Foundation, and patients and relatives known at the Radboudumc were also informed about this study by letter. Patients of thirteen years and older were asked to respond themselves. Relatives of patients under 13 years were asked to participate on behalf of them. After six weeks participants were reminded using the same channels as well as a week before closing the survey.

### Data analysis

We numbered the eight themes (displayed underlined in the text) in order of their prioritization given by the respondents. Additionally the respondents were asked to score the topics within a theme (displayed in *Italics* in the text). A score of 1 was given to the most important topic and the lowest score was equal to the total amount of topics within a theme. We stratified data on age groups of patients (0–5 years; 6–12 years; 13–17 years; 18–45 years; 46–65 years and 65 years and older), phenotype or clinical diagnose of the disorder as indicated by the participants (NS compared to the other NSSDs) and gender. Although we also asked for the gene mutation, 22% of the respondents answered that the gene mutation was not tested, identified or known. Due to this high number, we did not stratify on gene mutation.

Descriptive statistics (frequencies and percentages) were carried out to characterize demographics and the priorities in the themes and topics (mean and 95% confidence interval (ci)).

Statistical analyses were performed with SPSS 25. Whenever appropriate, we compared the priorities with the unpaired T-test or Mann-Whitney U test, and, for more than two groups, the Kruskal Wallis test. *P-*values < 0.05 were considered statistically significant. The significant difference between the consecutive prioritized topics within each theme are given and also significant differences between the first three topics within a theme and the consecutive topics. These results, together with the mean values of the scores of each topic, indicate the ranking of the topics within each theme as listed in its subsequent table. We used the same methodology to prioritize the themes.

Additionally significant differences in ranking are given, stratified according to: age groups, clinical diagnose (phenotype) (NS versus other NSSDs) and gender.

### Ethical approval and informed consent

Participation in this study guaranteed full anonymity and respect for the privacy of patients and confidentiality of the information provided. The process did not focus on substantive medical research and did not use patient invasive research methods. Although registered patients were also informed by the Radboudumc, the research was initiated and performed by the Dutch Noonan Syndrome Foundation. For this reason the Ethics Committee on Human Research of the Radboudumc pointed out that a formal CMO approval procedure was not necessary. All data were stored within a secured digital environment, conform standard practices and protocols at the research institutes involved.

## Results

### Demographics

A total of 80 respondents filled out the final questionnaire. The group of participating patients was small (n = 10; 12.5%) compared to the group of relatives (n = 70; 87.5%). 45% of the patients was male, 55% female. From the different NSSDs, the majority of respondents were (caregivers of) patients with NS (79%). The other characteristics are described in Table 1.

**Table 1:** Characteristics of the respondents

	N	%
**The person with a NSSD**		
Patient	10	12.5
Child from respondent	68	85.0
Family member (other than child)	2	2.5
	**80**	
**Gender of the person with NSSD**		
Woman	44	45.0
Man	36	55.0
**Age of person with NSSD**		
0–5 years	22	27.5
6–12 years	23	28.7
13–17 years	7	8.8
18–45 years	26	32.5
46–65 years	2	2.5
65 + years	0	0
**Type of NSSD**		
Noonan syndrome	63	78.8
Noonan syndrome with multiple lentigines	1	1.3
Noonan syndrome with loose anagen hair	7	8.7
Cardio-facio-cutaneous Syndrome	4	5.0
Costello syndrome	2	2.5
Not reported	3	3.7

### In general

Fifty-three topics in eight themes were identified by patients and relatives. The open section of the questionnaire did not result in additional topics. To compare the importance of the themes and topics to each other, the ranking of the themes related to each other, and the ranking of the topics within each theme are listed in the tables below.

### Prioritizing the themes

The first two themes had a statistical overlap in priority, indicating that according to the total group of respondents, theme 1 Physical problems (non-musculoskeletal related) and 2 Social, emotional and behavioral problems may be equally important. The priority of theme 1 differed significantly from theme 3 Problems with cognitive functioning and information processing (P < 0.05) and all lower prioritized themes, indicating that theme 1 may be the most important theme of the two first prioritized themes and thus of all the themes, according to the total group of respondents. The priority of the fourth prioritized theme Problems related to the musculoskeletal system differed significantly from the fifth prioritized theme Self-reliance and participation in society (P < 0.01) and all lower prioritized themes, indicating that the four physical and neuropsychological related themes are of more concern to the respondents than the four lower prioritized themes. The priority of the sixth prioritized theme Problems with the medical care system differed significantly from the as seventh prioritized theme Problems with fertility and desire to have children (P < 0.05) and the lower prioritized theme, theme 8 Familiarity with NSSDs. These results, together with the mean values of each theme, indicate that the themes can be ranked as listed in Table 2.

**Table 2:** Ranking of the eight themes according to importance

Rank	Theme	Number of topics within the theme	Mean	95% ci
1	Physical problems (non-musculoskeletal related)	10	2.71	2.33–3.09
2	Social, emotional and behavioral problems	6	3.00	2.59–3.41
3	Problems with cognitive functioning and information processing	5	3.33	2.92–3.74
4	Problems related to the musculoskeletal system	9	3.87	3.42–4.32
5	Self-reliance and participation in society	6	4.94	4.43–5.45
6	Problems with the care system	5	5.49	5.03–5.95
7	Problems with fertility and desire to have children	6	6.15	5.70–6.60
8	Unfamiliarity with NSSD	6	6.45	6.04–6.86

### Prioritizing the topics within each theme

#### Theme 1: Physical problems (non-musculoskeletal related)

The first three prioritized topics within this theme had a statistical overlap in priority. However looking more closely at the first three prioritized topics, the first prioritized topic *Coagulation problems* (e.g. nosebleeds, bruising, hematomas) was significantly more important than the fourth prioritized topic *Problems with eyesight* and all lower prioritized topics (P < 0.05). The second prioritized topic *Heart problems* was significantly more important than the seventh prioritized topic *Abnormal reaction to medication* and all lower prioritized topics (P < 0.05). The third prioritized topic *Feeding problems* was significantly more important than the eighth prioritized topic *Lymphatic problems* (P < 0.05). These results, together with the mean values of each topic, indicate that the topics within theme 1 can be ranked as listed in Table 3.

**Table 3:** The ranking of the 10 topics within the theme Physical problems (non-musculoskeletal related)

Rank	Topics of theme 1: Physical problems (non-musculoskeletal related)	Mean	95% ci
1	Coagulation problems	4.16	3.55–4.77
2	Heart problems	4.88	4.07–5.68
3	Feeding problems	5.01	4.40–5.63
4	Problems with eyesight	5.14	4.59–5.68
5	Problems with teeth	5.16	4.56–5.77
6	Metabolic and weight problems	5.19	4.53–5.85
7	Abnormal reaction to medication	5.67	5.15–6.19
8	Lymphatic problems	6.07	5.23–6.91
9	Ear, nose and throat problems	6.62	6.02–7.21
10	Skin problems	6.68	6.02–7.35

#### Theme 2: Social, emotional and behavioral problems

Within this theme, the first prioritized topic *Problems to identify and describe emotions experienced by one’s self and by others* was significantly more important than the third prioritized topic *Insecurities* (e.g. different physical appearance, feelings of not been taken seriously) and all lower prioritized topics (P < 0.01). The second prioritized topic *Anxiety and mood problems* was significantly more important than the fourth prioritized *topic Problems with social interactions* and all lower prioritized topics (p < 0.05). The third prioritized topic *Insecurities* was significantly more important than the fifth prioritized topic *Problems with social skills* and the lower prioritized topic, topic 6 *Behavioral problems* (P < 0.05). These results, together with the mean values of each topic, indicate that the topics within theme 2 can be ranked as listed in Table 4.

**Table 4:** The ranking of the six topics within the theme Social, emotional and behavioral problems

Rank	Topics of Theme 2: Social, emotional and behavioral problems	Mean	95% ci
1	Problems to identify and describe emotions experienced by one’s self and by others	2.75	2.42–3.08
2	Anxiety and mood problems	3.13	2.69–3.57
3	Insecurities	3.39	3.02–3.76
4	Problems with social interactions	3.79	3.44–4.14
5	Problems with social skills	3.93	3.55–4.31
6	Behavioral problems	3.93	3.44–4.42

#### Theme 3: Cognitive functioning and information processing

The first prioritized topic within this theme *Learning difficulties* (learning skills, adjustment to school, difficulties with school specific competences), was significantly more important than the second prioritized topic and all lower prioritized topics (p < 0.01). The second prioritized topic *Tempo of thinking and doing* was significantly more important than the fourth prioritized topic *Problems with planning and organizing* (p < 0.01). The third prioritized topic *Discrepancies in verbal and executive skills that can lead to overestimation of competences in daily life* was significantly more important than the fourth prioritized topic *Attention/concentration problems* (P < 0.05). These results, together with the mean values of each topic, indicate that the topics within theme 3 can be ranked as listed in Table 5.

**Table 5:** The ranking of the five topics within the theme Cognitive functioning and information processing

Rank	Topics of theme 3: Cognitive functioning and information processing	Mean	95% ci
1	Learning difficulties	2.25	1.95–2.55
2	Tempo of thinking and doing	2.78	2.52–3.04
3	Discrepancies in verbal and executive skills that can lead to overestimation in daily life	2.94	2.55–3.33
4	Problems with planning and organizing	3.38	3.10–3.66
5	Attention/concentration problems	3.59	3.26–3.92

#### Theme 4: Problems related to the musculoskeletal system

Within this theme, the first prioritized topic *Problems with motor skills* was significantly more important than the third prioritized topic *Problems with physical load (fatigue)* and all lower prioritized topics (P < 0.01). The second prioritized topic *Problems with growth and small stature* was significantly more important than the fourth prioritized topic *Problems with scoliosis/thorax deformity* and all lower prioritized topics(p < 0.05). The third prioritized topic *Problems with physical load (fatigue)* was significantly more important than the eighth prioritized topic *Fragile bones* (p < 0.01). These results, together with the mean values of each topic, indicate that the topics within theme 4 can be ranked as listed in Table 6.

**Table 6:** The ranking of the nine topics within the theme Problems related to the musculoskeletal system

Rank	Topics of theme 4: Problems related to the musculoskeletal system	Mean	95% ci
1	Problems with motor skills	3.58	3.11–4.05
2	Problems with growth and small stature	4.04	3.39–4.69
3	Problems with physical load (fatigue)	4.68	4.11–5.25
4	Problems with scoliosis/thorax deformity	4.89	4.37–5.41
5	Problems with physical mobility	5.06	4.47–5.65
6	Problems due to pain	5.07	4.47–5.67
7	Problems with fine motor skills	5.16	4.62–5.70
8	Fragile bones	5.78	5.18–6.38
9	Speech and articulation problems	6.44	5.80–7.08

#### Theme 5: Self-reliance and participation in society

The first five consecutive topics within this theme had a statistical overlap in priority, meaning that they were statistically equally important according to the total group of respondents. All these five topics were significantly more important than the as sixth prioritized topic *Difficulties with maintaining paid/volunteer work* (P < 0.01). This result, together with the mean values of each topic, indicate that the topics within theme 5 can be ranked as listed in Table 7.

**Table 7:** The ranking of the six topics within the theme Self-reliance and participation in society

Rank	Topics of theme 5: Self-reliance and participation in society	Mean	95% ci
1	Problems with keeping up at school	3.21	2.72–3.70
2	Problems with maintaining own control of care and life	3.25	2.93–3.57
3	Social isolation and loneliness	3.31	2.90–3.72
4	Problems with self-reliance, autonomy and independence	3.36	3.00-3.72
5	Difficulties finding paid/volunteer work	3.41	3.02–3.80
6	Difficulties with maintaining paid/volunteer work	4.32	3.93–4.71

#### Theme 6: Medical care system

Within this theme, the first two topics had a statistical overlap in priority, however the first prioritized topic *Unclear which problems are caused by the syndrome and which are person specific* and the second prioritized topic *NSSD specific care* were significantly more important than the third prioritized topic *Problems with managing the large amount of care and support* and all lower prioritized topics (P < 0.01). The third prioritized topic was significantly more important than the fourth prioritized topic *Problems finding support or help for the family* and the lower prioritized topics. (P < 0.01). These results, together with the mean values of each topic, indicate that the topics within theme 6 can be ranked as listed in Table 8.

**Table 8:** The ranking of the six topics within the theme Medical care system

Rank	Topics of theme 6: Medical care system	Mean	95% ci
1	Unclear which problems are caused by the syndrome and which are person specific	2.38	2.02–2.74
2	NSSD specific care	2.72	2.36–3.08
3	Problems with managing the large amount of care and support	2.96	2.66–3.26
4	Problems finding support or help for the family	4.01	3.66–4.36
5	Problems with directing fragmented care processes	4.19	3.83–4.55
6	Difficulties in communication with health care professionals	4.60	4.23–4.97

#### Theme 7: Fertility and desire to have children

The first prioritized topic *Hereditary causes and solutions of NSSD* within this theme, was significantly more important than the second prioritized topic *Prenatal diagnosis of NSSD* and all lower prioritized topics (p < 0.01). The second prioritized topic *Prenatal diagnosis of NSSD* was significantly more important than the fourth prioritized topic *Pre-implantation genetic diagnosis* and the lower prioritized topic (p < 0.05). The third prioritized topic *Prognosis in next generation* had statistical overlap with the lower prioritized topics. These results, together with the mean values of each topic, indicate that the topics within theme 7 can be ranked as listed in Table 9.

**Table 9:** The ranking of the five topics within the theme Fertility and desire to have children

Rank	Topics of theme 7: Fertility and desire to have children	Mean	95% ci
1	Hereditary causes and solutions of NSSD	2.03	1.73–2.33
2	Prenatal diagnosis of NSSD	2.81	2.55–3.07
3	Prognosis in a next generation	3.15	2.78–3.52
4	Pre-implantation genetic diagnosis	3.29	2.96–3.62
5	Reduced fertility	3.65	3.34–3.96

#### Theme 8: Familiarity with NSSDs

Looking at this theme, the first prioritized topic *More awareness of NSSD among health care professionals* was significantly more important than the second prioritized topic *More awareness at educational institutions* and all lower prioritized topics (p < 0.01). The second prioritized topic was significantly more important than the fourth prioritized topic *More understanding from social environment* and all lower prioritized topics (p < 0.05). The third prioritized topic *More awareness of NSSD in society* was significantly more important than the sixth prioritized topic *Problems finding resources to facilitate awareness with the syndrome among specific target groups* (P < 0.01). These results, together with the mean values of each topic, indicate that the topics within theme 8 can be ranked as listed in Table 10.

**Table 10:** The ranking of the six topics within the theme Familiarity with NSSDs

Rank	Topics of theme 8: Familiarity with NSSDs	Mean	95% ci
1	More awareness of NSSD among health care professionals	2.32	1.95–2.69
2	More awareness of NSSD at educational institutions	3.03	2.67–3.39
3	More awareness of NSSD in society	3.32	2.97–3.67
4	More understanding from social environment	3.36	2.97–3.75
5	Where to find suitable information about syndrome specific problems	3.67	3.30–4.04
6	Problems finding resources to facilitate awareness with the syndrome among specific target groups	4.82	4.48–5.16

### Age specific problems and needs

The prioritizations of some topics were different from the ranking of the total group of respondents, looking at the age groups (Table 11). Within theme 1 Physical problems (non-musculoskeletal related) three topics were ranked significantly different from the total group of respondents according to age group (Table 11). The topic *Feeding problems*, which was ranked on the third place in the total group, was prioritized on the first place in the group of children aged 0 to 5 and also in the group of children aged 6 to 12 years (p < 0.01). Also the topics *Metabolic and weight problems* and *Ear, nose and throat problems* were considered significantly more important by the youngest two age groups (both p < 0.05).

In theme 4 Problems related to the musculoskeletal system, the topic *Pain complaints* was significantly more important to elder patients than to young children (p < 0.05).

Also a clear age influence appeared in theme 5 Self-reliance and participation in society. The topic *Problems with keeping up at school* was more important to children and adolescents (p < 0.01) and *Finding and maintaining paid/volunteer work* was significantly more important to adults (p < 0.05).

**Table 11:** The ranking of topics within each theme that were significantly different according to age group

Theme	Topic	Mean (total number of topics within theme)	Ranking 0–5 y	Ranking 6–12 y	Ranking 13–17 y	Ranking 18–45 y	Ranking 46–65 y
1	Feeding problems	3 (10)	1	1	7	8	8
1	Metabolic and weight problems	5 (10)	3	3	8	6	10
1	Ear, nose and throat problems	9 (10)	7	8	10	10	8
4	Pain complaints	6 (7)	7	5	3	5	3
5	Problems with keeping up at school	1 (6)	1	1	5	6	6
5	Difficulties finding paid/volunteer work	5 (6)	5	5	1	2	3
5	Difficulties with maintaining paid/volunteer work	6 (6)	6	6	2	4	4

### NS specific problems compared to problems of the other NSSDs

Several topics were ranked significantly different for patients with NS than for patients with another NSSD (Table 12). Topics that were more important to individuals with NS compared to the other NSSDs were: *Heart problems* (Theme 1) (p < 0.05), *Problems with planning and organizing* (Theme 3) (p < 0.05) and *Pre-implantation genetic diagnosis* (Theme 7) (p < 0.05).

**Table 12:** The ranking of topics within each theme that were significantly different according to the clinical diagnose of NSSD (phenotype)

Theme	Topic	Mean (Total number of topics within theme)	Rank NS	Rank other NSSDs
1	Heart problems	2 (10)	2	8
1	Skin problems	10 (10)	10	3
3	Problems with planning and organizing	4 (5)	4	5
5	Self-reliance, autonomy and independence	4 (6)	5	1
7	Pre-implantation genetic diagnosis	4 (5)	3	4

Topics that were more important to patients with other NSSD were: *Skin problems* (Theme 1), *Self-reliance, autonomy and independence* (Theme 5) and *Finding support or help within the family* (Theme 6)(p < 0.05).

### Gender related problems

There were also several gender related problems (Table 13). To women the topics *Feeding problems* (Theme 1) (p < 0.05) and *Skin problems* (Theme 1)(p < 0.01) were more important compared to men, as well as *Social interactions* (Theme 2) (p < 0.05). To men the topic *Problems with social skills* (Theme 2) was more important (p < 0.05).

**Table 13:** The ranking of topics within each theme that were significantly different according to gender

Theme	Topic	Mean (Total number of topics within theme)	Ranking Female	Ranking Male
1	Feeding problems	3 (10)	2	7
1	Skin problems	10 (10)	8	10
2	Problems with social skills	5 (6)	6	4
2	Problems with social interactions	4 (6)	4	6

## Discussion

This is the first structured study to inventories and prioritize the problems and needs of patients with NSSDs and their relatives. Fifty three specific topics, clustered within eight themes, were actively mentioned by patients and their relatives as important issues to act on. Nineteen of these topics were related to strictly physical problems. Eleven topics were neuropsychological. The remaining 23 topics belonged to themes concerning care system and society (self-reliance, education and paid/volunteer work, care system, and familiarity) with the exception of the theme: *Fertility and desire to have children*.

The results show that the variety of issues patients with NSSDs and their relatives experience in daily life is very wide. This implicates that (the search for) solutions require just as much variety and should address all domains in life. Part of the high prioritized physical and neuropsychological topics were not surprising; they concern life threatening issues or have much impact on the quality of life, for example the topics *Coagulation problems* and *Heart problems*. Also, at the sixth and seventh international RASopathy symposia physical and neuropsychological issues were highlighted as the most urgent treatment targets [14, 15].

Despite many patient representative organizations working together with clinicians and researchers to share and discuss basic science, clinical issues and guideline development [11], there still seems to be a lack of awareness and limited collaboration [16]. This collaboration is very important to both know and acknowledge the needs of patients with NSSD and their relatives to be able to meet their needs in research, care as well as policy, both inside and outside health care institutions. We will discuss some of the prioritized topics in relation to the attention and priority they currently receive in research, medical care and policy and formulate some suggestions for potential solutions, without having the intention to be complete.

### Research

The themes Physical problems (non-musculoskeletal related) and Problems related to the musculoskeletal system were prioritized as first and fourth theme. Within the theme Physical problems (non-musculoskeletal related) *coagulation problems*, *heart problems* and *feeding problems* were prioritized as the first three topics. The fact that *coagulation problems* were prioritized as first topic, may partly be explained by the recent attention coagulation problems received at an annual meeting of the Dutch Noonan Syndrome Foundation.

Although coagulation problems have been studied much, there is no consensus how to diagnose, prevent and treat possible coagulation problems in patients with NSSD [17]. There is a huge amount of publications on diagnosis and treatment of heart problems in patients with and without NSSD. New therapeutical options, consisting of innovative medical Ras/MAPK pathway-inhibition treatment, have been introduced to manage patients with hypertrophic obstructive cardiomyopathy (and also severe lymphatic diseases) [18, 19]. However, efficiency and safety of this type of medical treatment is debated. Management of ERK1/2 or (cytosolic) pERK targets, causing, or contributing to NSSD specific pathologies may offer rational, specific targeting approaches. Future strategies may also emerge from the field of RAS-driven cancers. This is also the goal of the Advancing RAS/RASopathy Therapies (ART). This is an NCI-sponsored intramural and extramural collaboration for the study of RASopathies. ART aims to develop and study effective therapies and medical strategies to prevent clinical manifestations of NSSDs [20] .

The high prioritization of *Feeding problems* and *Metabolic and weight problems* among the younger groups of patients (and feeding problems among female gender) asks for more attention and focus. The huge problem of feeding problems in young children was also mentioned as an important topic in a recent survey of the European Medical Education Initiative on NS, in which they suggest to update the diagnostic criteria by Van der Burgt based on new clinical, molecular and endocrinological substantiations [1, 16, 21]. Little is known about the specific pathophysiology of the vomiting problems, although recently a new hypothesis has been developed in which the activated Ras/MAPK pathway plays an essential role [22, 23]. Also, little is known about the psychological and behavioral consequences in relation to the intake process of tube feeding, food refusal or the impact on the lives of the families combining the care of a patient with an NSSD with other responsibilities (e.g. work or the care for other children). The problems of vomiting in addition to feeding problems tend to have a high impact on both the (young) patient and the whole family. Vomiting may contribute to the development of Avoidance Restricted Food Intake Disorder (ARFID, DSM-V) [24]. More research on effective therapeutical options to control the vomiting may help prevent the development of (severe and long-term effects of) feeding problems.

A gender related difference was found in the importance of skin problems, indicating that gender related hormones or its importance could play a role in dermatological pathologies these patients experience. Strikingly skin problems were also mentioned as more important in patients with NSSDs other than NS. The pathophysiology of this phenomenon is still unknown. Within the theme Problems related to the musculoskeletal system the topics *Problems with motor skills*, *Problems with growth and small stature* and *Problems with physical load (fatigue)* were prioritized as the first three topics. These problems were not age, gender or type of NSSD dependent.

These results confirm findings of an earlier small study in which patients with NS reported particular problems related to pain, decreased muscle strength, fatigue, and clumsiness, which had an evident impact on functioning in daily life [25]. This could be objectively confirmed in children with NS [26]. Recently, the importance of possible neuropathic pain and neuropathies was suggested to be of importance in patients with NSSDs, leading to *Problems with motor skills* and *Problems with physical strain* and *Pain complaints*, the latter especially in the older age groups [27]. These findings indicate that more clinical and fundamental research is needed to help understand and manage NSSD specific problems related to the musculoskeletal system, e.g. the pathological effects of NSSD (the upregulation of RAS/MAPK/ERK components and pERK targets) in relation to pain and the development of neural, bone and muscle cells and tissue.

The themes Social, emotional and behavioral problems and Cognitive functioning and information processing were prioritized as the second and third theme. Within the theme Social, emotional and behavioral problems, the topic *Problems to identify and describe emotions experienced by one’s self and by others (alexithymia)* was given the highest priority, followed by *Anxiety and mood problems* and *Insecurities*. Within the theme Cognitive functioning and information processing, the topics *Learning difficulties*, *Tempo of thinking and doing a*nd *Discrepancies in verbal and executive skills that can lead to overestimation of competences in daily life* were given the highest priority.

Although neuropsychological functioning in NSSDs have been studied more extensively in the last decade, including alexithymia, still many issues should be addressed [28, 29]. This applies to both children and adults. Fortunately, recently studies have been performed to test the possibility and feasibility of social cognitive training programs [30, 31].

Better understanding of the pathological consequences of a pathogen mutation within the Ras/MAPK pathway may sometimes ask for a less fragmented and more integrated, multi-disciplinary approach, e.g. clinically and fundamentally. For example, problems that are more important to the other NSSD patients compared to NS patients may be partly explained by the suggestion that disorders caused by mutations in more downstream components of the Ras/MAP kinase pathway, may have a higher impact on dysregulation. Mutations in more proximal actors may receive more regulation levels, so the pathological effects may be compensated better [1]. In addition to searching for effective MEK-inhibitor options, management of pathway components and/or (cytosolic) pathological pERK targets (e.g. by managing cross-talk pathways) in specific cell types, tissues or organs may provide for new therapeutical options.

### Medical care

Physical and neuropsychological problems were highly prioritized. However, although there seems a high degree of overlap in medical care in different European countries, coordination between professionals involved in the management of NSSD patients, needs to be improved, as is the transition between pediatric and adult services and the use of medical guidelines [16]. It is often difficult to develop guidelines for patients with NSSD on evidence based medicine, as was stated in the recently published management guidelines of Costello syndrome [32]. These guidelines were based on expert opinion due to the lack of data for this rare condition.

*Coagulation problems* were prioritized as the most important of physical problems, even more important than *Heart problems*. This may partly be explained by the recent attention coagulation problems received at an annual meeting of the Dutch Noonan Syndrome Foundation. However, in a recent evaluation of bleeding disorders it was concluded that there is no current consensus on management of (possible) bleeding disorders in patients with NS [17]. Therefore it would be beneficial for patients to aim for clinical consensus and incorporate an update of recommendations into guidelines for clinical management.

In addition to a need for more research, the high prioritization of *Problems with motor skills* also implies a need for more NSSD specific medical care (such as physiotherapy or occupational therapy) and / or an update of the current clinical guidelines to better help manage problems related to the musculoskeletal system [25, 26].

*Heart problems* were significantly more important for patients and relatives of patients with NS, compared to other NSSDs. This may be due to other physical, neuropsychological and/or other themes related topics that are of equal or more concern to NSSD patients other than NS. In general, management of the most life threatening heart problems has been given much attention to and has improved over the last years. This may additionally explain why heart problems were not mentioned as the number one concern according to the total group of respondents. Also, the recent published survey on management of cardiac aspects in children with NS showed that medical care is appropriate [33]. However, the care needed in patients with NS and congenital heart disease using growth hormone is still unknown as are unmet medical needs [33].

The high prioritization of *Feeding related problems* indicate that current care according to general management principles, apparently falls short. As mentioned earlier, feeding problems were not mentioned in the diagnostic criteria of van der Burgt [21], although earlier studies described the prevalence of feeding problems in up to 76% of patients with NS [34, 35]. Our results and other studies underline a need for an update on the management and care of feeding related problems that specifically meet the needs of NSSD patients, especially of the youngest patients. In this context it is also important to mention the high prioritization of ear, nose and throat problems according to the younger age groups, compared to older patients. As there may be a relationship between infections in the oropharyngeal area and the development of feeding problems, this may ask for more specialized NSSD specific oropharyngeal attention in the care of these young patients [36]. Additionally, feeding problems were prioritized higher by female participants then by male. This may be explained by the observation that only female hyperactivated SHP2-D61A mutant mice resulted in increased energy expenditure and resistance to obesity in contrary to male mice, because of the synergistic action of estrogens [1, 37]. When children and adults have trouble with the intake of food, and additionally have a high energy expenditure [23, 38, 39] maintaining a healthy BMI may be even more challenging in female patients with NSSD than in male patients. This may explain this observed result. A recent evaluation study on feeding problems concluded that due to their high prevalence, it is necessary to evaluate and treat feeding problems in a multidisciplinary structured evidence-based way [36].

Another age specific result was the importance of *Pain complaints* that were more important to older patients than the other age groups, indicating that there is a need for NSSD specific management of pain [27].

Growth and small stature was also an important problem. However, patients were invited to participate in the questionnaire in January 2020 and shortly thereafter the Dutch Medicines Evaluation Board (College ter Beoordeling van Geneesmiddelen) approved growth hormone for the treatment of short stature due to NSSD (without problems with growth hormone). So growth and small stature now may be a less important problem.

Clinical genetic specialists should be aware of the highly prioritized pre-implantation genetic diagnostic concerns, especially of NS patients. Their concerns may be related to the better prognosis NS patients have compared to the non-NS group.

Although problems in adult patients can be severe, overall the intensity of the care and the clinical consequences of the problems seem to have the greatest impact on young patients. However, it is still not explicitly known how NSSDs impact the lives of adults and how medical care can and may change this [14, 16].

The prioritization of the themes Social, emotional and behavioral problems and Cognitive functioning and information processing show that neuropsychological issues are being considered almost equally important to the themes concerning physical problems. The current NS Clinical Management Guideline includes most of the mentioned social, emotional and behavioral topics, however the majority of the topics concerning cognitive functioning and information processing are not [40]. To optimize care of NSSD patients it would be beneficial to include these topics in a future edition of the NSSD clinical guideline.

Other striking findings concerning the care system are the need for *NSSD specific care* and *Problems with managing the amount of care and support* in different areas like care, school, speech therapy, behavior etc., prioritized second and third. This indicates that patients and their relatives need more support handling the huge amount of problems. Worth mentioning is the Dutch adaptation of NS clinical management guideline for insurance, legal and health care purposes, performed by Dutch health care researchers and patient representatives. The Dutch clinical guidelines includes a specific section to help patients how to manage the large amount of problems [13].

### Policy and society

Although NSSDs, with a prevalence of 1:1500 (which is half the amount of the prevalence of Down Syndrome) are not such rare diseases, in general it turns out to be difficult to generate more familiarity to a broader audience. To help solve this problem and to address all domains in life of patients with NSSD we want to give special attention on their needs outside the (medical) care and research institutes. In the 2010 management guideline of NS an individualized education plan for school-aged children is proposed, but no other specific guideline for non-medical care issues are mentioned [40].

The topics of the themes Social interaction, Cognitive functioning and information processing and Self-reliance and participation in society clearly state that there are major concerns related to these themes and ask for more focus and attention. What is striking is that *finding paid/volunteer work* is of significant more importance than *maintaining paid/volunteer work*. Help finding work for people with a chronic disease is a great challenge that needs intervention on government level. In the Netherlands there are middle and high schools specialized in educating children and adolescents with significant learning and behavioral challenges financed by the national government. These schools have small classes and when the school is well organized, maintains a close network of small and middle businesses in the local area that provide for subsidized jobs, training and coaching for this group of pupils. Challenges may actually be greater to children and adolescents with NSSD that have slightly less severe problems and have to attend normal middle and high schools with classes of 25–30 pupils and later to find a job without the help of a well-organized network of support.

Looking at theme 8, Familiarity with NSSDs, although prioritized last, is also a concern to patients with NSSD that should be addressed. More awareness of NSSD among health care professionals is ranked significantly first. This may be an indication that patients need additional tools to inform health care professionals in general, both inside and outside specialized clinics. There are some international sources of information available for patients and their relatives to help handle the most common physical, learning and behavioral problems. Several NS Teachers’ Guidelines have been developed by neuropsychological research institutes [41–43]. The information in these guidelines specifically underline NSSD related behavior and challenges. Developing international standards of NSSD specific teaching and other supportive tools may help generate essential information specific for caretakers of patients with NSSDs and support where it is currently needed.

### Strengths and limitations

This study is the first published study to systematically inventories and prioritize the needs of patients with NSSDs. The input and experiences of patients with NSSDs and their relatives are important information for professionals and other persons involved in their care and their lives. Although this study contains a fair number of respondents, most of them were relatives. Patients were underrepresented, as well as patients (and their relatives) of the older age groups. Consequently, it could be that relevant issues for patients (especially of the older age groups) have not been identified.

We stratified NS versus other NSSDs, because the different types of other NSSDs than NS separately did not generate a high enough number to stratify and analyze. This may be not the optimal choice, as CFC and CS often have a different presentation. However NSSDs other than NS in general tend to generate more severe pathologies then NS. So taking other NSSDs (than NS) as a group is not arbitrarily. Finally, as much literature has been published on Noonan syndrome and less on the other NSSDs, this choice of stratification created the opportunity to address five needs and problems specific to the group other than NS. This data may also indicate that the group NSSDs other than NS may have more in common than generally thought. This can be important information in medical care, research and policy.

Another limitation might be, that we have not stratified on gene mutation, because many participants mentioned that the gene mutation was not tested, identified or known.

Though we prioritized the themes and topics, our study does not provide information on how to prioritize the topics relative to each other, independent of the themes they were clustered in. Finally, international cooperation could help national patient representative organizations with restricted capacity and resources to provide for their patients’ needs more specifically and more easily.

### Future implications

The current organization of heath care and research is mostly symptom-directed and thereby most often scattered. Looking at the many indicated needs of this group of patients in a fragmented way might provide for limited solutions. Therefore it is important to consider what solutions would improve the quality of life in future the most.

The effectiveness and quality of medical treatment patients receive is determined by the (inter)national management guidelines and diagnostic and therapeutic innovations. Hence it is important to evaluate what patients in general feel is currently missing in current research, given care and their needs beyond research and health institutions.

This project provides insight in and answers to the needs patients and their relatives have into the process of research, care and policy development and evaluation. An example may be the option of an individual care plan, as a part of the (international) clinical guideline, which can help handle the great amount of health issues patients with NSSD have to handle.

New therapeutical options may emerge from management options of (cytosolic) pathological pERK targets and/or other pathway components (e.g. by managing cross-talk pathways) in specific cell types, tissues or organs, in addition to searching for effective MEK-inhibitor options. Evaluation studies show new insights concerning Ras/MAPK hyperactivation as a shared pathway, promising endocrine and homeostatic approaches, such as energy homeostasis (e.g. by leptin and insulin), calcium homeostasis and future treatments from the field of RAS-driven cancers. This asks for more (international) collaboration between institutions to develop more longitudinal patient cohort research to find which treatments are safe and effective.

There seems to be a need for more information to inform both health care and non-health care professionals like teachers, paramedics and non-professionals. Guidelines with cognitive, behavioral and social developmental characteristics and ways to subsistence, would help others to support patients in their development outside the doors of medical institutions. Such guidelines could give developmental support at young ages (at school e.g. teachers’ guidelines) and older ages (e.g. at work) and will help others to help patients achieving and maintaining independency in life.

It is difficult though, to achieve such goals alone. Therefor patient representative organizations are required to co-develop such tools and provide for effective implementation. Also fragmented health care is a problem patients cannot solve on their own. Government policy makers together with patient representing organizations should help to provide for future improvement. To achieve such goals, national and international collaboration between patient representing organizations would contribute to their empowerment and provide for more ways to meet their patients’ needs.

## Conclusions

This results underline the importance of methodologically inventorying the needs of NSSD patients, not only at the group level, but to also focus on specific needs according to e.g. age, phenotype and gender. For instance, it is remarkable that both the current Clinical Guidelines and the Noonan Syndrome diagnostic criteria give little to no attention to feeding problems, though our results indicate that, to the youngest patients, these problems have top priority. A similar situation appears to apply to the clinical management of e.g. coagulation, neuropsychological and musculoskeletal problems (like physiotherapy or occupational therapy) and to a need for (educational) tools to support patients inside and outside medical institutions, at school or at work.

Our study may help to shape targeted (clinical) management, research and policy and shed light on the complex phenotypes of NSSDs, the families’ and patients’ perspectives on the everyday consequences of the many different problems, as well as their needs.

## Data Availability

All data generated or analyzed during this study are included in this published article. Datasets are available via URL: https://www.radboudumc.nl/amalia-kinderziekenhuis/informatie-voor-professionals/leren-en-ontwikkelen and go to Contact, or from the corresponding author on reasonable request (jos.draaisma@radboudumc.nl).
